# Management of hematogenous osteomyelitis in children in Douala, Cameroon: diagnostic challenges, complications, and perspectives for improvement

**DOI:** 10.3389/fped.2026.1778153

**Published:** 2026-04-01

**Authors:** Pauline Mantho Fopa, Prosper Mouapi, Eric R. Bitchoka, Frédérique Azoo, Ritha Mbono, Théophile Kamguep, Jean Paul Engbang, Puis Fokam, Faustin Mouafo Tambo

**Affiliations:** 1 Faculty of Medicine and Pharmaceutical Sciences, University of Douala, Douala, Cameroon; 2 Pediatric Surgery Unit, Laquintinie Hospital of Douala, Douala, Cameroon; 3 General Surgery Unit, Gyneco-Obstetric and Pediatric Hospital of Douala, Douala, Cameroon; 4 Pediatric Emergency Unit, Laquintinie Hospital of Douala, Douala, Cameroon; 5 Pediatric Unit, Protestant Hospital of Ndogbati, Douala, Cameroon; 6 General Surgery Unit, Douala General Hospital, Douala, Cameroon; 7 Faculty of Medicine and Biomedical Sciences, University of Yaoundé I, Yaoundé, Cameroon

**Keywords:** hematogenous infection, pediatric osteomyelitis, prognostic factors, sickle cell disease, surgical management

## Abstract

**Background:**

Pediatric hematogenous osteomyelitis remains a major cause of morbidity in low-resource settings. In Douala, Cameroon, delayed diagnosis and comorbid conditions contribute to severe disease and unfavorable outcomes.

**Methods:**

We conducted a retrospective cross-sectional study of children aged 0–15 years diagnosed with hematogenous osteomyelitis in five referral hospitals in Douala between January 2017 and December 2024. Sociodemographic, clinical, microbiological, therapeutic, and outcome data were collected. Univariate analysis was performed using odds ratios (OR) with 95% confidence intervals (CI), and variables with *p* < 0.20 were entered into a multivariable logistic regression model to identify independent predictors of unfavorable outcome.

**Results:**

Among 306 pediatric osteoarticular infections, 102 were osteomyelitis and 81 met inclusion criteria. The mean age was 6.88 ± 3.98 years, with male predominance (sex ratio 1.79). Consultation was delayed beyond three months in 48.1% of cases. Blood cultures were positive in 21.6%, while focal cultures were positive in 71.1%, with *Staphylococcus aureus* as the predominant pathogen. Sickle cell disease (SS genotype) was identified in 25.9% of the cohort. Surgical management was required in 46.9% of patients, mainly in chronic forms. Complications occurred in 25.9% of cases. Independent predictors of unfavorable outcome included consultation delay >3 months (aOR 19.65), previous osteomyelitis (aOR 15.08), sickle cell disease (aOR 7.18), chronic osteomyelitis (aOR 4.54), functional impairment (aOR 2.99), abnormal ultrasound findings (aOR 3.69), and prolonged antibiotic therapy >3 months (aOR 14.88).

**Conclusion:**

Pediatric hematogenous osteomyelitis in Douala remains frequent and severe. Delayed consultation and sickle cell disease are major determinants of poor prognosis. Early diagnosis, strengthened microbiological capacity, and multidisciplinary management are essential to improve outcomes.

## Introduction

Pediatric osteomyelitis remains a significant cause of morbidity worldwide, particularly in low- and middle-income countries where delayed diagnosis and limited resources contribute to severe and chronic forms (1–3). Acute hematogenous osteomyelitis is the most common type in children and primarily affects the metaphyses of long bones.

While early diagnosis and targeted antibiotic therapy have improved outcomes in high-income settings (4), sub-Saharan Africa continues to face major challenges, including delayed consultation, limited microbiological facilities, and restricted access to specialized care (5–7). In Cameroon, severe and chronic forms remain frequent, often requiring prolonged antibiotic therapy and surgical intervention (8–10).

Surgical intervention is indicated in cases of abscess formation, sequestrum, or failure of medical therapy (11).

International guidelines recommend early empiric antibiotic therapy targeting *Staphylococcus aureus*, followed by treatment adaptation based on microbiological results, with surgery reserved for complicated or chronic cases (12, 13). *Staphylococcus aureus* remains the leading causative organism worldwide (14, 15). Nevertheless, adherence to these recommendations is challenging in many African settings, where limited resources and delayed diagnosis influence therapeutic decisions and outcomes.

In Cameroon, pediatric osteomyelitis remains a significant public health concern, with previous hospital-based studies reporting a high proportion of chronic and complicated forms at presentation, particularly in urban referral centers [Bahebeck et al., Mouafo et al.] (3, 4).

This study aimed to describe the epidemiological, clinical, microbiological, and therapeutic characteristics of pediatric hematogenous osteomyelitis and to identify predictors of unfavorable outcome in Douala.

## Methods

This retrospective cross-sectional study evaluated the management of pediatric osteomyelitis in Douala. It was conducted in five hospitals: Douala General Hospital, Laquintinie Hospital of Douala, Gyneco-Obstetric and Pediatric Hospital of Douala, Military Regional Hospital No. 2 of Douala, and Protestant Hospital of Ndogbati. Medical records of children aged 0–15 years diagnosed with hematogenous osteomyelitis between January 1, 2017, and December 31, 2024, were reviewed. Diagnosis was based on compatible clinical and radiological findings.

Hematogenous osteomyelitis was defined as infection occurring without preceding trauma, surgery, or open fracture, and supported by clinical and radiological findings consistent with hematogenous dissemination.

Demographic, clinical, microbiological, therapeutic, and outcome data were collected and analyzed using SPSS version 25. Univariate analysis was performed using odds ratios (OR) with 95% confidence intervals (CI). Variables with *p* < 0.20 were included in multivariable logistic regression to identify independent predictors of unfavorable outcome.

Ethical approval was obtained in accordance with the Declaration of Helsinki. Administrative authorization was granted by participating hospitals, and all data were anonymized.

## Results

A total of 306 pediatric osteoarticular infections were recorded during the study period, including 102 cases of osteomyelitis (33.3%). Eighty-one children met the inclusion criteria for hematogenous osteomyelitis.

**Figure 1:**
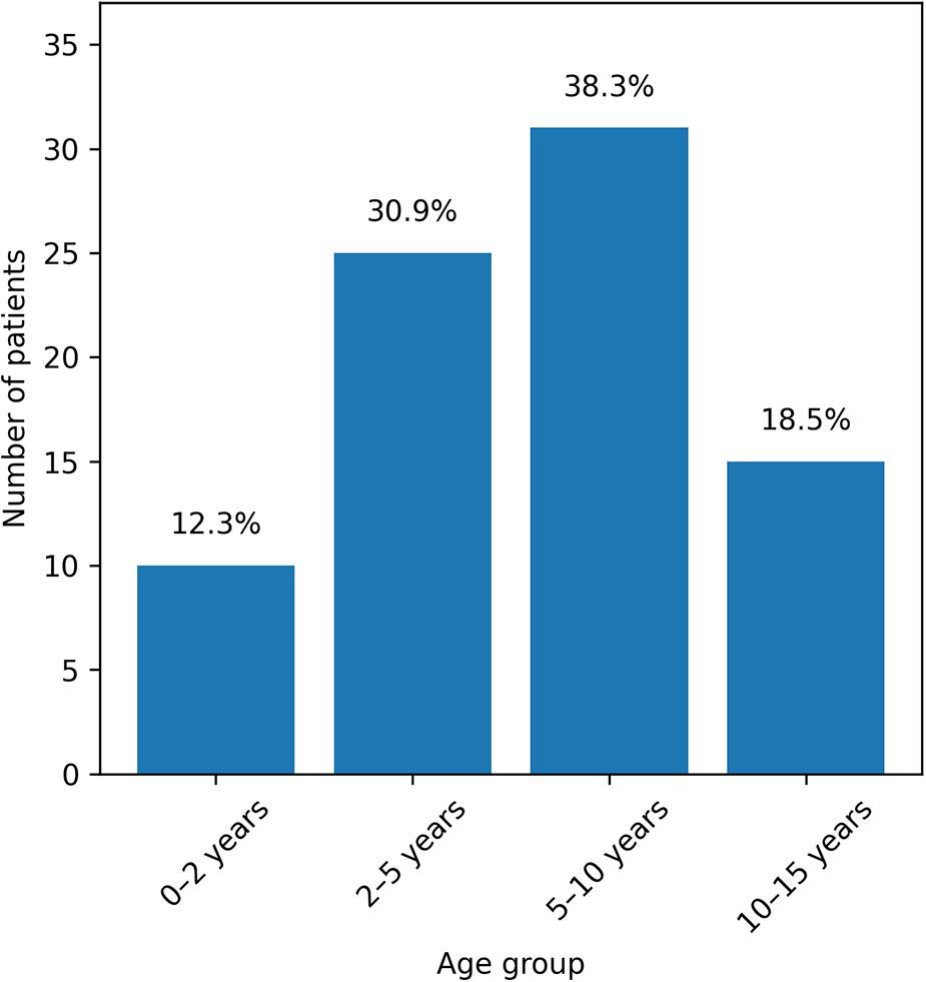
Distribution of patients by age group (*N* = 81).

The mean age (Figure 1) was 6.88 ± 3.98 years (range: 1 month–15 years), with a male-to-female ratio of 1.79. Consultation was delayed for three months in 39 patients (48.1%) (Table 1).

**Table 1:** Distribution of patients according to consultation delay.

Consultation delay (months)	Number (*N* = 81)	Percentage (%)
<1 month	31	38.3
1–3 months	11	13.6
>3 months	39	48.1
Total	**81**	**100**.**0**

Bold indicates the category with the highest frequency (most prevalent category).

Blood cultures were positive in 21.6%, while focal cultures were positive in 71.1%, with *Staphylococcus aureus* as the predominant pathogen (Tables 2, 3). Hemoglobin electrophoresis identified sickle cell disease (SS genotype) in 25.9% of the cohort.

**Table 2:** Microbiological findings.

Investigation	Performed *n* (%)	Positive *n* (%)	Main pathogens
Blood culture	60 (74.1)	13 (21.6)	*Staphylococcus aureus* (69.2%), *Salmonella spp.* (30.7%)
Focal culture	45 (55.6)	32 (71.1)	*Staphylococcus aureus* (90.6%), *Salmonella spp.* (9.4%)

**Table 3:** Distribution of isolated pathogens.

Pathogen	Blood cultures *n* (%)	Focal cultures *n* (%)
Staphylococcus aureus	9 (69.2)	29 (90.6)
Salmonella spp.	4 (30.7)	3 (9.4)

Surgical management (Table 4) was required in 46.9% of patients, mainly in chronic cases (85.7%). Complications (Table 5) occurred in 25.9%, most commonly joint stiffness. Sequestrectomy (Table 6) was the most frequently performed procedure (22/38; 57.9%). The delay to surgery was generally greater than one month, mainly due to preoperative optimization, particularly transfusion in sickle cell patients and financial constraints.

**Table 4:** Surgical management according to type of osteomyelitis.

Severity	Surgery yes (*N* = 38)	Surgery no	Total
Acute	–	25 (100.0%)	25 (100.0%)
Subacute	2 (14.3%)	12 (85.7%)	14 (100.0%)
Chronic	36 (85.7%)	6 (14.3%)	42 (100.0%)

**Table 5:** Distribution of patients according to complications. Overall complication rate was 25.9% (21/81).

Complications	Number (*N* = 21)	Percentage (%)
Joint stiffness	12	57.1
Persistent infection	7	33.3
Limb deformity	4	19.0
Pathological fracture	3	14.3
Paralysis	4	19.0
Residual pain	2	9.5
Death	1	4.7

**Table 6:** Surgical management characteristics.

Variable	*n* (*N* = 81)	%
Patients requiring surgery	38	46.9
Chronic osteomyelitis among surgical cases	36/42	85.7
Most frequent procedure: Sequestrectomy	22/38	57.9
Median delay to surgery	>1 month	–

In multivariable analysis (Table 7), independent predictors of unfavorable outcome were consultation delay >3 months (aOR 19.65), previous osteomyelitis (aOR 15.08), sickle cell disease (aOR 7.18), chronic osteomyelitis (aOR 4.54), functional impairment (aOR 2.99), abnormal ultrasound findings (aOR 3.69), and antibiotic duration >3 months (aOR 14.88).

**Table 7:** Multivariable logistic regression analysis of factors associated with unfavorable outcome.

Variable	Univariate OR (95% CI)	*P*-value	Adjusted OR (95% CI)	*P*-value
SIRS at presentation	5.60 (1.40–22.43)	0.009	–	–
Sickle cell disease	6.75 (2.59–25.18)	<0.001	7.18 (1.69–30.55)	0.0029
Previous osteomyelitis	14.17 (4.34–46.22)	<0.001	15.08 (3.80–59.75)	<0.001
Consultation delay >3 months	20.63 (4.09–103.87)	<0.001	19.65 (3.71–89.57)	<0.001
Functional impairment	2.99 (1.47–6.07)	<0.001	2.99 (1.47–6.07)	0.00012
Middle third bone involvement	6.46 (1.49–27.90)	0.005	–	–
Abnormal ultrasound findings	3.69 (1.81–12.53)	<0.001	3.69 (1.81–12.53)	0.00019
Chronic osteomyelitis	4.54 (1.26–23.58)	0.014	4.54 (1.26–23.58)	0.014
Antibiotic duration >3 months	14.88 (3.11–49.13)	<0.001	14.88 (3.11–49.13)	<0.001

## Discussion

This study provides an updated hospital-based overview of pediatric hematogenous osteomyelitis in Douala and identifies key determinants of unfavorable outcomes in a resource-limited urban setting.

Nearly half of the patients presented after more than three months of symptoms. Delayed consultation emerged as the strongest independent predictor of unfavorable outcome (aOR 19.65). This finding is consistent with previous reports from low-resource settings, where delayed diagnosis contributes to chronic evolution, bone destruction, and functional impairment (1, 16). In our context, financial constraints, reliance on traditional medicine, and limited access to specialized care likely contribute to this delay.

Microbiologically, *Staphylococcus aureus* was the predominant pathogen, in line with global epidemiological data (1, 14). The low positivity rate of blood cultures (21.6%) contrasts with the higher yield of focal samples (71.1%), a discrepancy commonly reported in settings with prior empirical antibiotic exposure and limited laboratory resources (15). These findings highlight the importance of improving microbiological capacity to enable targeted therapy and reduce prolonged empirical treatment.

Almost half of the patients required surgical intervention, predominantly in chronic forms. This proportion is comparable to previous Cameroonian reports (3). Surgical delay in our cohort was largely attributable to preoperative optimization—particularly in children with sickle cell disease—and financial barriers. Notably, no major postoperative complications were observed, suggesting that careful perioperative management may mitigate surgical risks even in vulnerable populations.

Sickle cell disease was independently associated with unfavorable outcome (aOR 7.18). This association is biologically plausible, as bone infarction, functional asplenia, and immune dysfunction predispose affected children to more severe and recurrent osteomyelitis (17). The identification of *Salmonella spp.* in a subset of cases further supports established epidemiological patterns in sickle cell populations (4). The high prevalence of sickle cell disease in our cohort underscores the need for multidisciplinary management integrating hematologic optimization.

Our findings are consistent with previous Cameroonian studies. Bahebeck et al. reported a high proportion of chronic hematogenous osteomyelitis and frequent surgical management, reflecting delayed presentation (3). Mouafo et al. emphasized the severity of osteomyelitis in children with sickle cell disease (4). However, our study extends these observations by providing multivariable analysis demonstrating that consultation delay, previous osteomyelitis, sickle cell disease, and chronic presentation are independent predictors of poor outcome.

Overall, delayed diagnosis, disease chronicity, and comorbid conditions remain the principal drivers of adverse outcomes in this setting. Strengthening early referral systems, improving microbiological diagnostic capacity, and promoting coordinated multidisciplinary care are essential strategies to improve prognosis in sub-Saharan Africa.

## Limitations

This study has several limitations. First, its retrospective design may have introduced information bias. Second, microbiological confirmation was not available for all patients, and blood cultures showed low positivity rates, likely due to prior antibiotic exposure. Third, hemoglobin electrophoresis was not performed in all patients, which may have led to underestimation of sickle cell disease prevalence. Finally, the hospital-based design may limit generalizability to rural populations.

## Conclusion

Pediatric hematogenous osteomyelitis remains a frequent and severe condition in Douala. Delayed consultation, previous osteomyelitis, sickle cell disease, and chronic presentation are major determinants of unfavorable outcomes. Improving early diagnosis, strengthening microbiological capacity, and ensuring multidisciplinary management—including optimized perioperative care—may significantly improve outcomes in resource-limited settings.

## Data Availability

The original contributions presented in the study are included in the article/Supplementary Material, further inquiries can be directed to the corresponding author.
